# Prognostic value of changes in quality of life scores in prostate cancer

**DOI:** 10.1186/1471-2490-13-32

**Published:** 2013-07-10

**Authors:** Digant Gupta, Donald P Braun, Edgar D Staren

**Affiliations:** 1 Cancer Treatment Centers of America®, 1336 Basswood Road, Schaumburg, IL 60173, USA

## Abstract

**Background:**

Several studies in the oncology literature have demonstrated the prognostic value of baseline quality of life (QoL). We investigated whether changes in QoL could predict survival in prostate cancer patients.

**Methods:**

We evaluated 250 prostate cancer patients treated at our institution between Jan 2001 and Dec 2009 who were available for a minimum follow-up of 3 months. QoL was evaluated at baseline and after 3 months of treatment initiation using EORTC-QLQ-C30. Cox regression evaluated the prognostic significance of baseline and changes in QoL scores after adjusting for relevant clinical and demographic variables.

**Results:**

Median overall survival was 89.1 months (95% CI: 56.5-121.7). Baseline QoL scale predictive of survival upon multivariate analysis was fatigue (p = 0.001). Associations between changes in QoL and survival, upon multivariate analysis, were observed for dyspnea and cognitive functioning. Every 10-point increase (worsening) in dyspnea was associated with a 16% increased risk of death (HR = 1.16; 95% CI = 1.02 to 1.30, p = 0.02), and every 10-point increase (improvement) in cognitive functioning was associated with a 24% decreased risk of death (HR = 0.76; 95% CI = 0.54 to 0.98, p = 0.04).

**Conclusions:**

This study provides preliminary evidence to indicate that prostate cancer patients with better baseline fatigue and patients whose dyspnea and cognitive functioning improves within 3 months of treatment are at a significantly decreased risk of mortality.

## Background

Prostate cancer is the most common cancer in men in the US [1-4]. It is also the second leading cause of cancer death among US men after lung cancer [5]. Each year in the US, approximately 220,000 new cases of prostate cancer are diagnosed, and 30,000 men die of the disease [6]. Global estimates suggest about 903,500 new cases and 258,400 deaths annually [7]. Despite its high morbidity, the etiology of prostate cancer remains largely unknown [5]. Tumor stage and Gleason score remain the two most powerful prognostic factors in prostate cancer [8]. Advanced prostate cancer is associated with significant morbidity, both from the underlying disease and associated co-morbidities, and also from treatment toxicity, which can profoundly affect patient quality of life (QoL) [9].

QoL is a multidimensional construct that includes physical, social, psychological and functional domains at the very least. Symptom palliation and QoL improvement have gained prominence in the assessment of new treatments, and the prolongation of life is no longer the sole aim of therapy [9]. This has led to the inclusion of QoL assessment as a primary endpoint in cancer clinical trials along with traditional endpoints of tumor response and survival. QoL measurements can provide information about the impact of the disease and its treatment to aid physicians in selecting both antineoplastic and supportive care therapy.

Baseline or pretreatment QoL has been shown to be a prognostic indicator in prostate cancer in previously published studies [10-13]. However, to the best of our knowledge, there is no study in the literature investigating the prognostic impact of changes in QoL scores on survival in prostate cancer, whether this is assessed at the time of initial cancer diagnosis or following disease relapse. In the current study, we investigated whether pretreatment QoL parameters as well as changes in QoL scores from baseline until 3 months after treatment initiation could predict survival in patients with prostate cancer. This study builds upon our previous work in this area investigating the relationship between changes in QoL and survival in other types of cancers [14-16].

## Methods

### Study population

We examined 250 histologically confirmed prostate cancer patients treated at Cancer Treatment Centers of America® (CTCA) at Midwestern (MRMC) and Southwestern (SRMC) Regional Medical Centers between Jan 2001 and Dec 2009. The inclusion criteria for participation in this study were a histological diagnosis of prostate cancer and the ability to read English. Patients with all stages of prostate cancer were eligible for the study. Patients were excluded if they were unable to give informed consent or were unable to understand or cooperate with study conditions.

A trained clinical coordinator was responsible for determining eligibility, describing the study, and obtaining informed consent. All patients were assured that refusal to participate would not affect their future care in any way. Patients who chose to participate were presented with the questionnaire at their initial visit and instructed to return their completed questionnaires to the clinical coordinator within 24 hours. Thus, patients completed questionnaires prior to receiving therapy at our facility.

Additional patient data recorded for this study was age, stage of disease at diagnosis, prior treatment history (previously treated versus newly diagnosed) and treatment received at our institution. The only follow-up information required was the date of death or the date of last contact/last known to be alive, obtained from the tumor registries at Midwestern and Southwestern Regional Medical Centers. This study was approved by the Institutional Review Board at CTCA.

### QoL assessment

European Organization for the Research and Treatment of Cancer Quality of Life Questionnaire (QLQ-C30) was used to assess patient QoL. It emphasizes a patient’s capacity to fulfill the activities of daily living. It is a 30-item cancer specific questionnaire that incorporates five functioning scales (physical, role, cognition, emotional, and social), eight symptom scales (fatigue, pain, and nausea/vomiting, dyspnea, insomnia, loss of appetite, constipation, diarrhea), financial well-being scale and a global scale (based on two items: “how would you rate your overall health during the past week” and “how would you rate your overall quality of life during the past week”). The raw scores are linearly transformed to give standard scores in the range of 0–100 for each of the functioning and symptom scales. Higher scores in the global and functioning scales and lower scores in the symptom scales indicate better QoL. A difference of 5–10 points in the scores represents a small change, 10–20 points a moderate change and greater than 20 points a large, clinically significant change from the patient’s perspective [17]. This instrument has been extensively tested for reliability and validity [18-20]. QoL was assessed at baseline and after 3 months of treatment initiation at our institution.

### Statistical analysis

Patient survival was the primary end point and defined as the time interval between the date of first patient visit to the hospital and the date of death from any cause or the date of last contact/last known to be alive. Two separate analyses were performed. First, the relationship between baseline QoL and patient survival was investigated for 250 patients. Second, the relationship between change in QoL scores between baseline and 3 months and survival was assessed for the same patient cohort. Change scores were calculated by subtracting the baseline scores from the 3-month QoL scores. The overall survival was calculated using the Kaplan-Meier method. Clinical and QoL variables were evaluated using univariate Cox proportional hazards models to determine which parameters showed individual prognostic value for survival. Multivariate Cox proportional hazards models were then performed to evaluate the joint prognostic significance of all QoL and clinical factors. Each QLQ-C30 scale was treated as a continuous variable for the purpose of Cox regression analyses. The effect of QoL parameters on patient survival was expressed as hazard ratios (HRs) with 95% confidence intervals (CIs). Changes of 10 or more points on a 0 to 100 scale are considered clinically relevant [21], therefore, we present hazard ratios for a 10-point change on the continuous QoL variables. An effect was considered to be statistically significant if the p value was less than or equal to 0.05. All statistical tests were two-sided. All data were analyzed using IBM SPSS version 20.0 (IBM, Armonk, NY, USA).

Cox regression with time-independent covariates assumes that the ratio of hazards for any two groups remains constant in proportion over time. We checked this assumption by examining log-minus-log plots for the categorical predictors. For continuous predictors, this assumption was checked using an extended Cox model with time-dependent covariates. Potential multicollinearity was assessed in two steps. Large values (above 0.75) of Pearson’s correlation coefficients were used as an initial screen for pairs of QoL variables. As a second check, the variance inflation factor was used with the final model to verify that multicollinearity was not significantly influencing model coefficients [22,23]. In order to minimize instability of the final multivariate model resulting from high multicollinearity, global QoL was evaluated separately because it is most highly correlated with all other variables on the QLQ-C30 questionnaire, and also because it is difficult to interpret and manipulate clinically. Finally, to assess the possible influence of sample bias on the results as well as to investigate the stability of the model coefficients, we performed a bias-corrected and accelerated (BCa) bootstrap resampling procedure. We generated 1000 samples, each the same size as the original data set, by random selection with replacement. Cox regression was then run separately on these 1000 samples to obtain robust estimates of the standard errors of coefficients, and hence the p values and 95% BCa confidence intervals of the model coefficients [24].

## Results

### Patient characteristics

Table 1 describes the baseline characteristics of our patient cohort. At the time of this analysis, 39 deaths had occurred among the 250 participants. Majority of the patients were newly diagnosed with stage II disease. The proportion of patients receiving chemotherapy, radiotherapy, hormone therapy and surgery at our institution were 12%, 74.8%, 66% and 1.2% respectively. Chemotherapy received at our institution was highly correlated with prior treatment history such that 4% of newly diagnosed versus 23.8% of previously treated patients received chemotherapy (chi-square p <0.001). Similarly, 85.9% of newly diagnosed versus 58.4% of previously treated patients received radiotherapy at our institution (chi-square p <0.001).

**Table 1:** Baseline characteristics of 250 prostate cancer patients

**Characteristic**	**Categories**	**Number**	**Percent**
Age at diagnosis (years)	▪ Mean	59.7	
	▪ Median	59.0	
	▪ Range	37-79	
Vital status	▪ Death	39	15.6
	▪ Alive	211	84.4
Treatment history	▪ Newly diagnosed	149	59.6
	▪ Previously treated	101	40.4
Stage at diagnosis	▪ Stage I	3	1.2
	▪ Stage II	168	67.2
	▪ Stage III	31	12.4
	▪ Stage IV	48	19.2
Chemotherapy at CTCA	▪ No	220	88.0
	▪ Yes	30	12.0
Radiotherapy at CTCA	▪ No	63	25.2
	▪ Yes	187	74.8
Hormone therapy at CTCA	▪ No	85	34.0
	▪ Yes	165	66.0
Surgery at CTCA	▪ No	247	98.8
	▪ Yes	3	1.2

Table 2 displays the baseline QoL scores across the two categories of prior treatment history. Patients with newly diagnosed disease had statistically significantly better QoL compared to patients who were previously treated for all QoL scales except for insomnia, diarrhea and financial scales.

**Table 2:** Baseline quality of life scores stratified by prior treatment history

**Baseline variable**	**Newly diagnosed**	**Previously treated**	**P**
	**(N = 149)**	**(N = 101)**	
	**Mean (standard deviation)**	**Mean (standard deviation)**	
**General quality of life**			
Global	70.7 (23.6)	58.4 (27.1)	<0.001*
**General function**			
Physical	88.5 (17.9)	79.0 (23.2)	<0.001*
Role	86.9 (24.5)	74.0 (31.3)	<0.001*
Emotional	77.0 (22.7)	70.7 (24.2)	0.04*
Cognitive	85.3 (21.4)	79.2 (22.6)	0.03*
Social	85.1 (24.2)	73.1 (29.6)	0.001*
**General symptom**			
Fatigue	23.4 (25.9)	36.4 (26.2)	<0.001*
Nausea/Vomiting	4.2 (12.2)	8.0 (17.4)	0.04*
Pain	16.5 (23.6)	31.1 (32.7)	<0.001*
Dyspnea	12.7 (21.0)	18.4 (23.7)	0.04*
Insomnia	27.2 (28.2)	32.0 (30.8)	0.21
Appetite loss	10.1 (21.1)	16.1 (25.2)	0.04*
Constipation	8.0 (18.4)	16.1 (25.6)	0.004*
Diarrhea	8.5 (18.2)	8.5 (19.2)	0.97
Financial	17.7 (29.0)	25.4 (31.6)	0.09

*P < 0.05.

Table 3 describes the results of univariate Cox regression analysis for baseline patient characteristics. Treatment history and stage at diagnosis were significantly associated with survival while age was not. Median overall survival for the entire patient cohort was 89.1 months (95% CI: 56.5-121.7 months). The median survival for newly diagnosed and previously treated disease was 82.7 and 41.3 months respectively, log-rank p <0.001. The median survival for stages I-II and stages III-IV was 81.9 and 48.0 months respectively, log-rank p <0.001.

**Table 3:** Baseline characteristics and associated HRs for death

**Characteristic**	**HR (95% CI)**	**P**
Age at diagnosis (years) used as continuous variable*	1.28 (0.87 – 1.72)	0.18
Stage at diagnosis (stages I-II as reference)	3.28 (1.71 – 6.30)	<0.001
Treatment history (newly diagnosed as reference)	8.32 (4.15 – 16.65)	<0.001

*HRs correspond to a 10-point increment for age.

### Association between baseline quality of life and survival

Table 4 describes the baseline scores for all dimensions of EORTC QLQ-C30 instrument. Among the EORTC QLQ-C30 functioning scales, emotional functioning had the lowest (worst) mean score of 74.5 while the highest (best) mean score of 84.6 was recorded for physical functioning. Among the EORTC QLQ-C30 symptom scales, nausea/vomiting had the lowest (best) mean score of 5.8 while the highest (worst) mean score of 29.2 was recorded for insomnia. Table 4 also displays the results of univariate and multivariate Cox regression analyses for each QoL variable. The HRs along with their 95% CIs for every 10-point increase in all EORTC QLQ-C30 scales are given. On univariate analysis, several baseline QoL variables were predictive of survival: global, physical, role, fatigue, pain, appetite loss and constipation. Before proceeding with multivariate analysis, we checked the bivariate Pearson’s correlation among the QoL variables to screen for observable multicollinearity. All correlation coefficients were smaller than the pre-decided cut-off level of r = 0.75. As a result, all QoL variables were considered in the multivariate analysis. On multivariate analysis, after adjusting for prior treatment history and stage at diagnosis, only fatigue was found to be statistically significantly associated with survival. A separate multivariate model was run for global QoL after adjusting for prior treatment history and stage at diagnosis. Global QoL was not found to be significantly associated with survival. VIF values for baseline QoL variables ranged from 1.1 (diarrhea) to 4.0 (fatigue), none of which indicates a significant problem with multicollinearity [22,23]. There was no evidence of non-proportional hazards in the multivariate models presented.

**Table 4:** Baseline QoL measures and associated HRs for death

**Baseline variable**	**QoL score mean (SD)**	**Univariate**		**Multivariate**	
		**HR (95% CI)**	**P**	**HR (95% CI)**	**P**
**General quality of life**					
Global	65.8 (25.7)	0.85 (0.74 – 0.96)	0.007*	0.92 (0.80 – 1.04)	0.19
**General function**					
Physical	84.6 (20.7)	0.81 (0.70 – 0.92)	0.001*	0.83 (0.64 – 1.03)	0.09
Role	81.7 (28.1)	0.87 (0.78 – 0.96)	0.006*	1.12 (0.88 – 1.37)	0.32
Emotional	74.5 (23.5)	0.98 (0.85 – 1.11)	0.78	0.97 (0.73 – 1.22)	0.84
Cognitive	82.8 (22.1)	1.02 (0.88 – 1.17)	0.74	1.36 (0.99 – 1.51)	0.07
Social	80.2 (27.1)	0.90 (0.80 – 1.01)	0.08	1.04 (0.84 – 1.24)	0.72
**General symptom**					
Fatigue	28.6 (26.8)	1.16 (1.06 – 1.27)	0.002*	1.45 (1.19 – 1.73)	0.001*
Nausea/Vomiting	5.8 (14.6)	1.12 (0.98 – 1.26)	0.08	0.88 (0.55 – 1.23)	0.50
Pain	22.4 (28.5)	1.16 (1.06 – 1.26)	0.001*	1.09 (0.92 – 1.26)	0.31
Dyspnea	15.0 (22.3)	0.98 (0.83 – 1.13)	0.78	0.88 (0.71 – 1.05)	0.17
Insomnia	29.2 (29.3)	1.01 (0.90 – 1.13)	0.82	0.99 (0.82 – 1.16)	0.89
Appetite loss	12.5 (23.0)	1.11 (1.01– 1.22)	0.04*	0.98 (0.75 – 1.21)	0.85
Constipation	11.3 (21.9)	1.18 (1.07 – 1.28)	0.001*	1.12 (0.96 – 1.27)	0.13
Diarrhea	8.5 (18.6)	0.98 (0.82 – 1.15)	0.85	1.02 (0.85 – 1.20)	0.79
Financial	21.4 (30.2)	1.04 (0.95 – 1.14)	0.36	0.91 (0.74 – 1.08)	0.27

• HRs correspond to a 10-point increment for QoL scores.

• 2 sets of multivariate models were constructed: one for global QoL and other for all general function and symptom variables combined.

• Multivariate model (for general function and symptom variables combined) adjusted for prior treatment history, stage at diagnosis and all baseline QoL variables.

• Multivariate model for global QoL adjusted for prior treatment history and stage at diagnosis.

• *P < 0.05.

In order to further investigate the stability of the classical multivariate Cox models reported in Table 4, we conducted a bootstrap resampling procedure based on 1000 samples. The bootstrap estimates of the multivariate HRs along with corresponding p values and 95% BCa CIs were calculated. We found no significant differences in regression coefficients and their corresponding p values between the classical Cox regression and bootstrap Cox regression models (results not shown in the interest of space).

### Association between changes in quality of life and survival

Table 5 describes the change in scores from baseline to 3 months for all dimensions of EORTC QLQ-C30 instrument. On an average, they were small. Table 5 also displays the results of univariate and multivariate Cox regression analyses for change in QoL scores. On univariate analysis, no change variable was significantly predictive of survival. Before proceeding with multivariate analysis, we checked the bivariate Pearson’s correlation among the change scores to screen for observable multicollinearity. All correlation coefficients were smaller than the pre-decided cut-off level of r=0.75. As a result, all QoL change variables were considered in the multivariate analysis. On multivariate analysis, after adjusting for prior treatment history and stage at diagnosis, the change variables that were significantly predictive of survival were cognitive functioning and dyspnea. A separate multivariate model was run for change in global QoL after adjusting for prior treatment history and stage at diagnosis. Change in global score was not found to be significantly associated with survival. VIF values for change in QoL variables ranged from 1.1 (change in diarrhea) to 3.3 (change in fatigue), none of which indicates a significant problem with multicollinearity. There was no evidence of non-proportional hazards in the multivariate models presented.

**Table 5:** Change in QoL measures and associated HRs for death

**Change variable**	**QoL change mean (SD)**	**Univariate**		**Multivariate**	
		**HR (95% CI)**	**P**	**HR (95% CI)**	**P**
**General quality of life**					
Global	−1.9 (28.3)	1.01 (0.90 – 1.12)	0.80	0.96 (0.85 – 1.07)	0.46
**General function**					
Physical	−2.0 (22.7)	1.10 (0.98 – 1.23)	0.09	1.15 (0.93 – 1.37)	0.19
Role	−4.3 (33.4)	1.07 (0.99 – 1.16)	0.09	0.92 (0.75 – 1.08)	0.31
Emotional	3.8 (25.2)	0.95 (0.84 – 1.06)	0.36	0.91 (0.71 – 1.12)	0.39
Cognitive	1.1 (24.6)	0.92 (0.80 – 1.04)	0.16	0.76 (0.54 – 0.98)	0.04*
Social	−1.6 (31.9)	1.06 (0.97 – 1.15)	0.19	1.03 (0.87 – 1.20)	0.71
**General symptom**					
Fatigue	2.8 (29.7)	0.94 (0.84 – 1.05)	0.28	0.97 (0.94 – 1.20)	0.77
Nausea/Vomiting	2.8 (22.3)	0.95 (0.82 – 1.08)	0.45	0.77 (0.56 – 1.03)	0.06
Pain	0.6 (31.0)	0.94 (0.84 – 1.04)	0.24	0.91 (0.73 – 1.09)	0.34
Dyspnea	1.2 (26.7)	1.09 (0.98 – 1.19)	0.09	1.16 (1.02 – 1.30)	0.02*
Insomnia	−2.5 (34.0)	0.96 (0.87 – 1.05)	0.36	0.92 (0.79 – 1.04)	0.19
Appetite loss	0.9 (28.2)	1.01 (0.90 – 1.12)	0.82	1.03 (0.87 – 1.20)	0.68
Constipation	0.6 (25.6)	0.94 (0.82 – 1.05)	0.28	0.97 (0.82 – 1.12)	0.69
Diarrhea	2.0 (25.5)	0.99 (0.87 – 1.11)	0.88	0.94 (0.80 – 1.08)	0.38
Financial	3.4 (34.9)	1.0 (0.91 – 1.08)	0.93	1.08 (0.97 – 1.20)	0.17

• HRs correspond to a 10-point increment for QoL scores.

• 2 sets of multivariate models were constructed: one for global QoL and other for all general function and symptom variables combined.

• Multivariate model (for change in general function and symptom variables combined) adjusted for prior treatment history, stage at diagnosis and all QoL change variables.

• Multivariate model for change in global QoL adjusted for prior treatment history and stage at diagnosis.

• *P <0.05.

In order to further investigate the stability of the classical multivariate Cox models reported in Table 5, we conducted a bootstrap resampling procedure based on 1000 samples. The bootstrap estimates of the multivariate HRs along with corresponding p values and 95% BCa CIs were calculated. We found no significant differences in regression coefficients and their corresponding p values between the classical Cox regression and bootstrap Cox regression models (results not shown in the interest of space).

We decided to explore the data further by conducting separate multivariate analysis for newly diagnosed and previously treated patients. In newly diagnosed patients, none of the change variables was significantly predictive of survival. However, in previously treated patients, cognitive functioning was significantly predictive of survival such that every 10-point increase (improvement) in cognitive functioning from baseline to 3 months was associated with a 41% decreased risk of death (HR = 0.59; 95% CI = 0.27 to 0.93, p = 0.02).

## Discussion

In this study, we found that prostate cancer patients with better baseline fatigue and patients whose dyspnea and cognitive functioning improves within 3 months of treatment are at a significantly decreased risk of mortality. The association between baseline QoL and survival in prostate cancer has been investigated and reported in a few previously published studies [10-13].

A pooled analysis of data from three RCT’s of metastatic hormone-resistant prostate cancer (HRPC) indicated that bone scan result (P < 0.0001), hemoglobin level (P < 0.0001), performance status (P = 0.0322), insomnia (P = 0.002) and appetite loss (P = 0.0015) were independent predictors of survival [10]. A study by Sullivan et al. in metastatic HRPC reported that patients with better baseline HRQL have better predicted survival, time to disease progression and pain prognosis than those with worse HRQL. Change in HRQL (12-week) improved the predictive accuracy for most clinical outcomes [13]. Lis et al. found that baseline patient satisfaction with health and physical subscale, psychological and spiritual subscale, family subscale, and overall HRQL are predictive of survival in patients with prostate cancer. After adjusting for the effects of treatment history and Gleason score, patient satisfaction with health and physical subscale was found to be significantly associated with survival (P = 0.04) [12]. In an analysis of data from three randomized phase III multicenter trials, Halabi et al. reported statistically significant association between pain and survival in castration-refractory prostate cancer (CRPC). The median survival times were 17.6 months (95% CI, 16.1- 19.1 months) and 10.2 months (95% CI, 8.6- 11.3 months; P <.001) in men with low (<17) and high (≥ 17) pain scores, respectively [11]. Our current study takes the previous research in this area to the next level by examining the impact of changes in QoL scores (as opposed to simply assessing the baseline QoL scores) on survival in prostate cancer patients undergoing treatment.

The results of this study have important implications for both clinical and research practices. They suggest that baseline QoL should be considered when planning treatment and regular QoL assessment performed during the course of treatment. Furthermore, interventions aimed at improving specific QoL parameters should be applied when indicated. The utility of this approach to patient management, based on the findings described in this study, would be validated definitively if interventions that enhance specific QoL parameters are shown to enhance survival.

Thus, the findings reported here suggest that QoL monitoring, coupled with treatment to improve fatigue, dyspnea and cognitive function when indicated, should be investigated in prospective studies in prostate cancer. Positive effects on survival as a consequence of interventions designed specifically to improve patient symptoms and QoL independent of tumor therapy would go a long way towards establishing causative relationships between specific QoL parameters and disease control. Although some progress has been made with respect to the treatment of fatigue in cancer patients, clinical effectiveness is inconsistent and unpredictable. And there are at present no effective means to address more complex QoL factors such as global health. This challenges the cancer research enterprise to develop greater understanding of the complex physiology responsible for all aspects of QoL, and to use this information to develop more effective and predictable methods to favorably modulate this critical aspect of patient health and wellness.

Several limitations of this study need to be acknowledged. Our study, because of its retrospective nature, relies on data not collected to test a specific hypothesis. As a result, we could not control for certain factors in our analyses that could influence survival such as treatment received at our institution, medical co-morbidities, socioeconomic factors, support system, exercise and educational level. The patient cohort was limited only to those patients who were English speakers and therefore is not representative of the complete spectrum of prostate cancer patients. A majority of our patients had stage II disease at presentation to our hospital. As a result, we acknowledge that our findings may not be applicable to advanced-stage prostate cancer patients, an issue that needs to be tested in suitable patient populations. Moreover, this study does not reveal a causative relationship between QoL and survival. Rather, patient QoL was found to act as a surrogate marker for otherwise undetected prognostic factors [25]. QoL scores were assessed over a 3-month interval only which may not be sufficient time for score changes to develop in other QoL parameters that may be prognostic of survival. We did not control for the multiple comparisons made in this study, but this is acceptable for hypothesis-generating studies [26]. Finally, we also think that restricting the analysis to newly diagnosed patients (patients with no prior treatment history) would have been more accurate, as it would have allowed for evaluation of true overall survival time, that is, time from the date of diagnosis to the date of death. However, doing so would have caused a significant reduction in the sample size. In our study, the survival time was calculated from the day of first visit at our hospital because QoL information was not available at the time of diagnosis for previously treated patients.

This study also has several strengths, including no missing data on any EORTC QLQ-C30 variables for the entire study sample; the use of a valid and reliable QoL instrument; the availability of clinical parameters in nearly all patients; and availability of mature and reliable survival data. As is the case for all exploratory retrospective studies, the most important outcome that can be achieved is the development of a hypothesis suggested by the results. As a consequence of this study, we hypothesize that the parameters of fatigue, dyspnea and cognitive function are independent determinants of survival in prostate cancer, and should be regularly assessed and when indicated, targeted for intervention.

## Conclusions

This exploratory study provides preliminary evidence to indicate that prostate cancer patients with better baseline fatigue and patients whose dyspnea and cognitive functioning improves within 3 months of treatment have a significantly increased probability of survival. Given that QoL is as meaningful as the actual length of life in patients with prostate cancer, these findings should be used in clinical practice to systematically address QoL-related problems of prostate cancer patients throughout their treatment course.

## Competing interests

The authors declare that they have no competing interests.

## Authors’ contributions

DG and DPB participated in concept, design, data collection, data analysis, data interpretation and writing. EDS participated in concept, design, data interpretation and writing. All authors read and approved the final manuscript.

## Pre-publication history

The pre-publication history for this paper can be accessed here:

http://www.biomedcentral.com/1471-2490/13/32/prepub
